# Arachidonic Acid as an Early Indicator of Inflammation during Non-Alcoholic Fatty Liver Disease Development

**DOI:** 10.3390/biom10081133

**Published:** 2020-07-31

**Authors:** Klaudia Sztolsztener, Adrian Chabowski, Ewa Harasim-Symbor, Patrycja Bielawiec, Karolina Konstantynowicz-Nowicka

**Affiliations:** Department of Physiology, Medical University of Bialystok, 15-089 Bialystok, Poland; adrian@umb.edu.pl (A.C.); eharasim@umb.edu.pl (E.H.-S.); patrycja.bielawiec@umb.edu.pl (P.B.); karolina.konstantynowicz@umb.edu.pl (K.K.-N.)

**Keywords:** arachidonic acid, inflammation, inflammatory precursor, lipid accumulation, oxidative stress, non-alcoholic fatty liver disease

## Abstract

Non-alcoholic fatty liver disease (NAFLD) is a chronic liver disease characterized by excessive lipid deposition. Lipid metabolism disturbances are possibly associated with hepatocyte inflammation development and oxidative balance impairment. The aim of our experiment was to examine the first moment when changes in plasma and liver arachidonic acid (AA) levels as a pro-inflammatory precursor may occur during high-fat diet (HFD)-induced NAFLD development. Wistar rats were fed a diet rich in fat for five weeks, and after each week, inflammation and redox balance parameters were evaluated in the liver. The AA contents in lipid fractions were assessed by gas–liquid chromatography (GLC). Protein expression relevant to inflammatory and lipogenesis pathways was determined by immunoblotting. The oxidative system indicators were determined with assay kits. Our results revealed that a high-fat diet promoted an increase in AA levels, especially in the phospholipid (PL) fraction. Importantly, rapid inflammation development via increased inflammatory enzyme expression, elevated lipid peroxidation product content and oxidative system impairment was caused by the HFD as early as the first week of the experiment. Based on these results, we may postulate that changes in AA content may be an early indicator of inflammation and irreversible changes in NAFLD progression.

## 1. Introduction

Non-alcoholic fatty liver disease (NAFLD) is one of the most common chronic liver diseases in Western societies [1,2]. Today, it is estimated that nearly 25% of the global population might be suffering from NAFLD [1,3,4]. This condition is characterized by histological changes resulting from excessive lipid deposition in more than 5% of the liver cell volume without the inordinate consumption of alcohol [1,5,6]. In nearly all Western countries, the major causes of the liver disease’s occurrence that may be distinguished are high-calorie and high-fat diets. Moreover, recent studies confirmed a significant association between the progression of NAFLD and the increasing number of type 2 diabetes and obesity cases [6,7]. In the sera of obese patients with coexisting fatty liver, there are significant changes in the levels of adiponectin, leptin and resistin. These molecules, together with pro-inflammatory cytokines, released from the adipose tissue can be involved in hepatic steatosis development [8].

The development of NAFLD often begins with mild fatty liver changes and may progress to non-alcoholic steatohepatitis (NASH), characterized by inflammation accompanying excessive lipid accumulation, a condition that is reversible. However, progressive NASH leads to more severe irreversible changes, such as the development of liver fibrosis, cirrhosis and finally liver failure or hepatocellular carcinoma (HCC) [1,2,4,5,6].

Prolonged, increased dietary fat consumption, exceeding the energy requirements of the body, results in the excessive accumulation of lipids in hepatocytes, mitochondrial dysfunction and oxidative balance impairment in the liver. The excessive deposition of lipids primarily in the triacylglycerol (TAG) fraction may initiate a cascade of changes leading to the increased accumulation of diacylglycerols (DAGs) and ceramides (CERs) [2,9]. The excessive accumulation of these lipid fractions may cause an impairment in lipid metabolism, an inflammatory response and the evolution of oxidative stress [10,11]. What is most important in the pathophysiology of NAFLD is not only the quantities of the major lipid classes accumulating in the liver, which are crucial for steatosis deterioration, but also the composition of the fatty acids in these fractions [10]. Interestingly, the excessive accumulation of free fatty acids (FFAs) can determine the sensitization of the liver to a series of “hits”, thereby leading to steatosis and the development of NASH [1].

Overnutrition and, especially, high-fat feeding serve as an exogenous source of arachidonic acid (AA, C20:4), which belongs to the n-6 polyunsaturated fatty acids (PUFAs) and is associated with metabolic disorders. An increased supply of PUFAs can cause the intensified generation of pro-inflammatory lipid compounds such as arachidonic acid and AA derivatives, contributing to cellular inflammatory signaling [2]. Oxidative stress and associated inflammation play a key role in the development and progression of NAFLD. It has long been recognized that arachidonic acid is a precursor of pro-inflammatory compounds also known as eicosanoids, i.e., prostaglandins, leukotrienes and thromboxane A2, whose increased deposition may cause NAFLD deterioration [2,12].

As previously noted, it is important to determine the time point when the inflammation and associated irreversible changes develop in the hepatocytes. Changes in the activity of enzymes responsible for the oxidative stress pathway are good indicators of when medical intervention should be implemented. Therefore, the following study aims to determine changes in lipid composition during NAFLD development. The data presented herein concern the differences in the content of AA in selected lipid fractions in high-fat diet (HFD)-induced NAFLD at different time points, which is considered to play the main role in the progression of oxidative stress and inflammation occurrence. Thus, our study will investigate the potential relationship between AA content and inflammation occurrence during non-alcoholic fatty liver disease development.

## 2. Materials and Methods

### 2.1. Experimental Model

The experiment was carried out on male Wistar rats initially weighing 100–150 g and that were housed in standard holding conditions (temperature of 22 ± 2 °C, with a reverse light–dark cycle of 12/12 h) with unrestricted access to water and food. To ensure that the animals did not compensate by eating or drinking less, the amount of water and food was monitored throughout the experiment. Then, after a one-week period of acclimatization, all the rats were divided into two groups: (1) a control group (*n* = 6 in each group) fed with a basal diet with reduced fat-calorie content (57.1% carbohydrates, 30.5% protein and 12.4% fat; ∑ saturated: 36.0%, ∑ MUFA: 24.7%, ∑ PUFA: 39.3%), (2) a high-fat diet group (*n* = 6 per group in each week of the diet) fed a diet rich in fatty acids (the energy content of the high-fat diet was distributed as follows: 60% fat, 20% carbohydrates and 20% protein; ∑ saturated: 72.0%, ∑ MUFA: 16.8%, ∑ PUFA: 11.2%; overall calories: 5.21 kcal/g). The high-fat diet was supplied by the Research Diets Inc. (New Brunswick, NJ, USA, cat no. D12492), and the fatty acid composition of the experimental diet was determined by gas–liquid chromatography (GLC). All values are expressed as percentages of the total molar amount of fatty acids. Moreover, two control groups were distinguished: the first in the 1st week and the second in the 5th week, both receiving standard rodent chow. On the basis of no considerable changes being observed upon the same assessment between the two control groups, the results were referred to as being from one control group (0 week). Fasted overnight animals, at the end of the experiment, at different time points (1, 2, 3, 4 and 5 weeks) were anaesthetized by the intraperitoneal injection of pentobarbital (80 mg/kg of body weight) and euthanized by bleeding them to death. From the inferior vena cava, the blood was collected into heparinized tubes and then centrifuged to obtain plasma, which was used in analysis. The liver tissue was promptly removed and frozen in liquid nitrogen using pre-cooled aluminum tongs and stored at −80 °C until measurements. The animal study was consistent with the guidelines approved by the Ethics Committee on Animal Care at the Medical University of Bialystok (28 May 2008, approval number: 32/2008).

### 2.2. Liver Histological Analysis

For analysis, the specific parts of the liver lobes from each rat were fixed in a 10% aqueous formaldehyde solution and then were dehydrated in a series of alcohols. Thereby, the prepared liver tissues were embedded in paraffin. Sections (4 μm thick) were stained with hematoxylin and eosin (H + E) as described by Konstantynowicz-Nowicka et al. [13].

The prepared histological slides were evaluated under a light Olympus BX41 microscope equipped with an Olympus DP12 camera (200× magnification; 20× lens, 10× eyepiece; Hamburg, Germany) by three independent pathologists. In our previous study [13], the same tissues were evaluated for NAFLD occurrence with the use of the NAFLD activity score (NAS) system, which showed that NAFLD occurred after 3 weeks of a HFD.

### 2.3. Analysis of the Liver and Plasma Lipid Contents

The individual fatty acid methyl esters in the obtained plasma and liver samples were extracted with a solution of chloroform/methanol (2:1, *v/v*) (the method of Folch) [14] and separated into fractions—DAGs, TAGs, FFAs and phospholipids (PLs)—using thin-layer chromatography (TLC) [15]. The separated fractions were transmethylated with a 14% methanol solution of boron trifluoride and quantitated in accordance with the retention times of standards using gas-liquid chromatography—GLC (Hewlett Packard 5890 Series II Gas Chromatograph; Agilent Technologies, CA, USA; containing a capillary column and flame ionization detector—HP-INNOWax)—as described previously in detail [15]. Based on the fatty acid composition, the arachidonic acid content in the particular lipid fraction was expressed in nanomoles per gram of tissue.

### 2.4. Immunoblotting

In brief, Western blotting was used to determine the expression of proteins involved in: eicosanoid and prostanoid synthesis pathways—cyclooxygenase-1 (COX-1), cyclooxygenase-2 (COX-2) and 15-lipooxygenase (15-LO) with antibodies from Santa Cruz Biotechnology, Inc., Dallas, TX, USA, and nuclear factor erythroid 2-related factor 2 (Nrf-2) with an antibody from Abcam, Cambridge, UK; lipogenesis—diacylglycerol acyltransferase 1 (DGAT1) with an antibody from Novus Biotechnologicals, Centennial, CO, USA, and diacylglycerol acyltransferase 2 (DGAT2) with an antibody from Santa Cruz Biotechnology, Inc.; inflammatory processes—nuclear factor-κβ (NF-κβ) and transforming growth factor β (TGF-β) with antibodies from Cell Signaling, and interleunkin-6 (IL-6) with an antibody from Abcam as previously described by Konstantynowicz-Nowicka et al. [16]. Due to the assessment of both high- and low-weight proteins, a wet and semi-dry transfer system was used. All the proteins were electrophoretically separated on 10% TGX Stain-Free Precast Gels (BioRad, Warsaw, Poland) then transferred to nitrocellulose or polyvinylidene fluoride (PVDF) membranes, depending on the transfer method. After blocking in TTBS buffer containing 5% nonfat dry milk or 5% bovine serum albumin (BSA), the membranes were immunoblotted with primary antibody and probed with appropriate horseradish peroxidase-conjugated secondary antibodies.

The determination of the total protein concentration in the liver tissue was performed with the use of a bicinchoninic acid (BCA) protein assay kit with BSA as a standard. Densitometric analysis of the immunoblotting signal was performed with a ChemiDoc visualization system (BioRad). The expression of the analyzed proteins was standardized to the total protein expression, and the control was set at 100%.

### 2.5. Determination of the Oxidative and Antioxidative Parameters

Prior to the determination of oxidative stress components, the liver tissue was homogenized in a radioimmunoprecipitation assay (RIPA) buffer (25 mg of tissue/250 µL of buffer) for malonyldialdehyde (MDA); in ice-cold phosphate buffer saline (PBS) at 20 mg of tissue/1 mL of PBS for superoxide dismutase 2 (SOD2), catalase (CAT) and total antioxidant capacity (TAC); and in that at 10 mg of tissue/90 µL of PBS for 4-hydroxynonenal (4-HNE) and advanced glycation end-product (AGE) determinations. Then, we centrifuged the obtained homogenates in RIPA buffer at 1,600× *g* at 4 °C for 10 min to quantify MDA. In order to quantify CAT, SOD2 and TAC, homogenates in PBS were centrifuged at 12,000× *g* at 4 °C for 5 min, and samples were centrifuged at 5,000× *g* at 4 °C for 5 min to assess 4-HNE and AGEs. Then, the supernatant fractions were relocated into separate tubes and stored at −80 °C for further analysis.

The liver’s concentrations of CAT and SOD2 were determined with commercial ELISA kits from Cloud-Clone Corp. (Houston, TX, USA). The absorbance of these biomarkers was measured spectrophotometrically at 450 nm by the use of a microplate reader (Synergy H1 Hybrid Reader, BioTek). Finally, the oxidative stress enzyme concentrations were calculated from the obtained standard curves. The results are expressed in picograms and nanograms per milligram of tissue for SOD2 and CAT, respectively.

For the quantitative determination of the MDA content in the liver homogenates, we used the thiobarbituric acid reactive substances (TBARS) assay kit from Cayman Chemical Company (Ann Arbor, MI, USA). This method is based on the reaction of MDA binding with thiobarbituric acid (TBA), and the resulting MDA–TBA adducts were measured colorimetrically at 530 nm. Next, the MDA values were calculated and are expressed in nanomoles per milligram of tissue.

To determine 4-HNE and AGE concentrations, we used ELISA kits from Biorbyt (Cambridge, UK). The absorbance was assessed spectrophotometrically at 450 nm. Then, the oxidative component concentrations were obtained from standard curves. The expressed amounts of 4-HNE and AGE are reported as picograms and nanograms per milligram of tissue, respectively.

Moreover, the parameter of the TAC of the liver tissue was determined by the use of a colorimetric TAC assay kit (Abcam). The absorbance was measured spectrophotometrically at 570 nm. Then, the parameter was calculated according to the manufacturer’s protocols and is expressed as nanomoles per milligram of tissue.

### 2.6. Data Analysis

The data from experiment are expressed as the mean ± standard deviation. Our statistical analysis was carried out using GraphPad Prism 5 (California, USA). The assumptions of the methods used in the analysis (normality of the data distribution and homogeneity of the variance) were checked using the Shapiro–Wilk test and Bartlett’s test. Statistical differences between groups were examined by one-way test ANOVA followed by an appropriate post-hoc test (Tukey’s test and *t*-test). For all the data, a *p*-value < 0.05 was accepted as statistically significant.

## 3. Results

### 3.1. Changes in the Liver Histology

**Figure 1:**
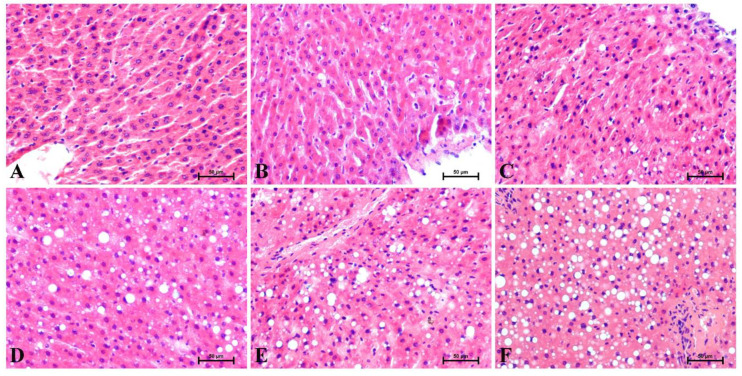
Representative hematoxylin and eosin (H + E) stains of the liver sections at the individual time points from (**A**) 0 week, as the control group, and the experimental HFD weeks: (**B**) 1, (**C**) 2, (**D**) 3, (**E**) 4 and (**F**) 5. The results from each rat (*n* = 6) were collected as independent determinations of the specific parts of the liver lobes. 200× magnification. On the lower right, the calibration bar equals 50 μm.

Overall, in Figure 1 are shown representative histological images of the H + E-stained liver sections. Histological alterations were evaluated by three independent pathologists. Five-week high-fat feeding affected the level of lipid deposits in the liver, and increased hepatocyte ballooning was observed. In the control group (0 week), after H + E staining were observed radially arranged hepatic trabeculae without ballooning degeneration (Figure 1A). All evaluated sections at the end of the experiment at the different time points (1, 2, 3, 4 and 5 weeks) showed slight to severe steatosis with a linear increase in balloon degeneration (Figure 1B–E) as compared to the control group. With each consecutive week of our study, the steatosis took on a more severe course. The pathologists noticed visible disorganization of the liver parenchyma in response to high-fat feeding. The highest grade of steatosis and ballooning occurred at the fifth week of HFD feeding (Figure 1F).

### 3.2. Changes in the Arachidonic Acid Content in the Liver and Plasma

**Figure 2:**
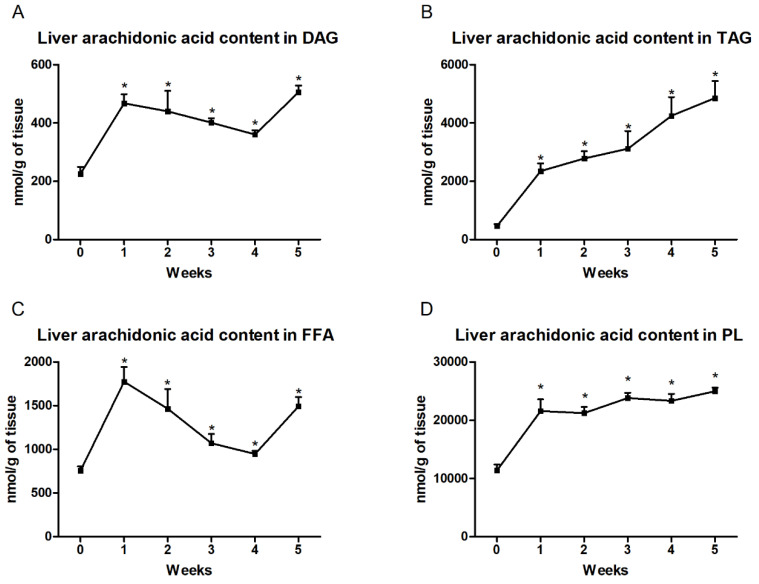
Liver contents of arachidonic acid in the (**A**) diacylglycerol (DAG), (**B**) triacylglycerol (TAG), (**C**) free fatty acid (FFA) and (**D**) phospholipid (PL) fractions. Changes in the arachidonic acid content in the selected lipid fractions were examined by the gas–liquid chromatography (GLC) method. The data are expressed as mean ± SD and are based on six independent determinations. * *p* < 0.05, significant difference compared with the control group (0 week).

In the liver, we observed significantly increased arachidonic acid content in the DAG fraction at each week in the high-fat feeding group (first week: +107.2%; second week: +94.9%; third week: +77.5%; fourth week: +59.8%; fifth week: +123.8%; *p* < 0.001; Figure 2A) compared to the control group. Moreover, in the TAG fraction, the level of AA gradually increased in all the examined groups and these changes were statistically significant (first week: +405.2%; second week: +498.7%; third week: +571.4%; fourth week: +815.0%; fifth week: +945.5%; *p* < 0.001; Figure 2B) in comparison with the control group. In the each week of high-fat feeding, we also found significantly increased arachidonic acid content in the FFAs (first week: +134.7%; second week: +93.7%; third week: +41.6%; fourth week: +25.2%; fifth week: +97.4%; *p* < 0.001; Figure 2C) and PLs (first week: +89.5%; second week: +86.4%; third week: +108.9%; fourth week: +104.9%; fifth week: +119.3%; *p* < 0.0001; Figure 2D) from that in the control group.

**Figure 3:**
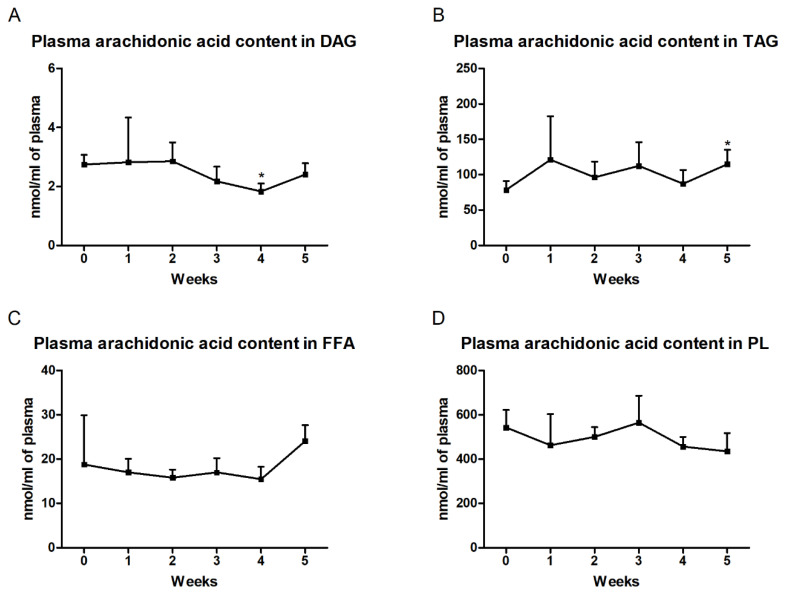
Plasma contents of arachidonic acid in the (**A**) diacylglycerol (DAG), (**B**) triacylglycerol (TAG), (**C**) free fatty acid (FFA) and (**D**) phospholipid (PL) fractions. Changes in the arachidonic acid content in the selected lipid fractions were examined by the gas–liquid chromatography (GLC) method. The data are expressed as mean ± SD and are based on six independent determinations. * *p* < 0.05, significant difference compared with the control group (0 week).

In the plasma, we observed markedly reduced arachidonic acid content in the DAG fraction only in the fourth week (fourth week: −33.2%; *p* < 0.01; Figure 3A). Furthermore, we also noticed an increased level of AA in TAG at the end of our study (fifth week: +46.4%; *p* < 0.05; Figure 3B). In the FFA and PL fractions, no significant changes in the arachidonic acid concentration during HFD feeding were found from that in the control group (Figure 3C,D).

### 3.3. Changes in the Expression of Proteins Involved in Eicosanoid and Prostanoid Synthesis Pathways

**Figure 4:**
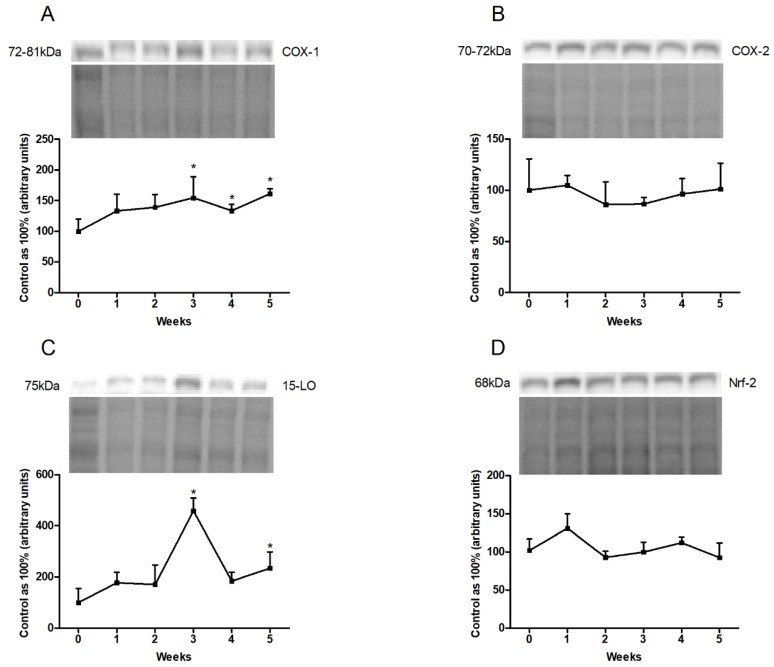
Representative Western blots illustrating the expression of proteins from eicosanoid and prostanoid synthesis pathways, i.e., (**A**) cyclooxygenase-1 (COX-1), (**B**) cyclooxygenase-2 (COX-2), (**C**) 15-lipoxygenase (15-LO) and (**D**) nuclear factor erythroid 2—related factor 2 (Nrf-2) in the liver homogenates. The protein expression was determined by the Western blotting method, as described in detail in the Materials and Methods section. The data are expressed as mean ± SD and are based on six independent determinations. * *p* < 0.05, significant difference compared with the control group (0 week).

The COX-1 expression in the liver homogenates showed a significant rise at the end of our study (third week: +54.5%, *p* < 0.05; fourth week: +33.4%, *p* < 0.05; fifth week: +61.3%, *p* < 0.01; Figure 4A). In the case of 15-LO expression, a substantial increase in the third and fifth weeks of high-fat feeding (third week: +358.8%, *p* < 0.001; fifth week: +134.9%, *p* < 0.05; Figure 4D) was observed. However, COX-2 and Nrf-2 expression remained unchanged in each week of high-fat diet from that in the control group (Figure 4B,C).

### 3.4. Changes in the Expression of Inflammatory Pathway Proteins

**Figure 5:**
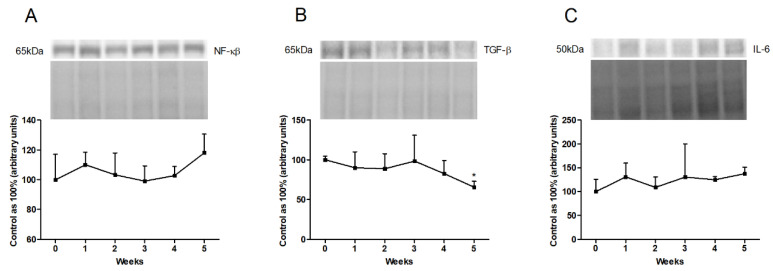
Representative Western blots illustrating the expression of inflammatory pathway proteins, i.e., (**A**) nuclear factor-κβ (NF-κβ), (**B**) transforming growth factor β (TGF-β) and (**C**) interleukin-6 (IL-6) in the liver homogenates. The protein expression was determined by the Western blotting method, as described in detail in the Materials and Methods section. The data are expressed as mean ± SD and are based on six independent determinations. * *p* < 0.05, significant difference compared with the control group (0 week).

In the liver homogenates, we showed a trend toward an increase in the expression of NF-κβ at the end of the experiment (fifth week: *p* = 0.1380; Figure 5A). Furthermore, the high-fat feeding resulted in a reduced expression of TGF-β in the last week of our study (fifth week: −34.5%, *p* < 0.001; Figure 5B). We also observed a trend toward to an increase in IL-6 expression in the fifth week of the HFD (fifth week: *p* = 0.0886; Figure 5C).

### 3.5. Changes in the Expression of Proteins Directly Involved in Lipogenesis Pathway

**Figure 6:**
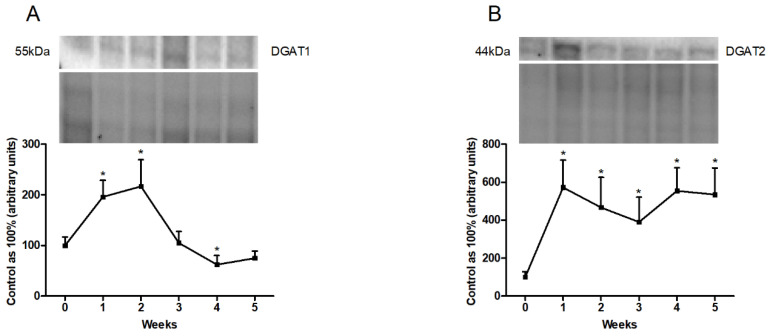
Representative Western blots illustrating the expression of proteins involved in lipogenesis, i.e., (**A**) diacylglycerol acyltransferase 1 (DGAT1) and (**B**) diacylglycerol acyltransferase 2 (DGAT2) in the liver homogenates. The protein expression was determined by the Western blotting method, as described in detail in the Materials and Methods section. The data are expressed as mean ± SD and are based on six independent determinations. * *p* < 0.05, significant difference compared with the control group (0 week).

In the liver homogenates, the total expression of DGAT1 increased significantly in the first and second weeks (first week: +95.8%; second week: +116.8%; *p* < 0.01; Figure 6A). By contrast, the HFD provoked a significant decrease in the fourth week (fourth week: −37.5%; *p* < 0.05; Figure 6A) as compared with the expression in the standard chow-fed group. In addition, we observed a substantial rise in DGAT2 expression in all the experimental model groups (first week: +472.9%; *p* < 0.01; second week: +279.2%; third week: +358.7%; *p* < 0.05; fourth week: +454.7%; fifth week: +434.9%; *p* < 0.01; Figure 6B).

### 3.6. Changes in the Oxidative and Antioxidative Parameters

**Figure 7:**
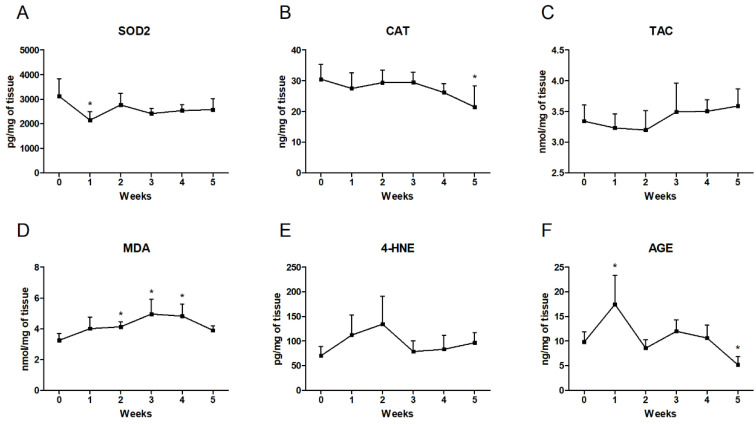
Modulation of the oxidant and antioxidant indicators, i.e., (**A**) superoxide dismutase 2 (SOD2), (**B**) catalase (CAT), (**C**) total antioxidant capacity (TAC), (**D**) malonyldialdehyde (MDA), (**E**) 4-hydroxynonenal (4-HNE) and (**F**) advanced glycation end-products (AGEs) in the liver homogenates. The indicator levels were determined using assay kits, as described in detail in the Materials and Methods section. The data are expressed as mean ± SD and are based on six independent determinations. * *p* < 0.05, significant difference compared with the control group (0 week).

The high-fat feeding induced a lower hepatic SOD2 concentration at the beginning of our experiment (first week: −31.1%; *p* < 0.05; Figure 7A) than in the standard chow-fed group. In addition, rats in the high-fat administration group exhibited significantly decreased hepatic catalase values only in the last week of the experiment (fifth week: −29.6%; *p* < 0.05; Figure 7B). To our surprise, we found no significant differences in the total antioxidant capacity between the HFD and the control groups at any time of the experiment (Figure 7C). In the liver homogenates, the level of MDA increased substantially in the second, third and fourth weeks of the HFD (second week: +27.1%; third week: +52.5%; fourth week: +48.7%; *p* < 0.01; Figure 7D). During the high-fat chow administration, we observed a trend towards to an increase in the 4-HNE content in the first two weeks of the HFD (first week: *p* = 01204; second week: *p* = 0.0810; Figure 7E). In the case of AGE concentration, we noticed a considerable increment in the first week of the HFD (first week: +77.6%; *p* < 0.05; Figure 7F) and a considerable reduction at the end of our experiment (fifth week: −47.3%; *p* < 0.01; Figure 7F) from the concentration in the control group.

## 4. Discussion

It has long been recognized that NAFLD is a common liver disease in Western civilizations. The major factor that influences and alters lipid metabolism and, as a consequence, causes abnormal lipid storage, is excessive nutrition rich in fat. An enhanced supply of fatty acids causes their increased accumulation in the cytoplasm of hepatocytes, mainly as triacylglycerols. Through histological assessment, we revealed time-dependent changes in the quantity and sizes of lipid droplets formed in the liver tissue. It is still unclear whether TAG deposition is the liver’s protective mechanism against lipotoxicity or is a culprit in NAFLD development [17]. We suspect that lipid accumulation to a certain point protects against the enhanced supply of fatty acids. After exceeding 5% of hepatocyte volume, the deposition of TAG in the liver possibly exhibits lipotoxic effects. Increased lipid storage in hepatocytes leads to the development of hepatic steatosis, which may be the cause of inflammation, and then contributes to progression into NASH [18]. This is in line with the time-dependent effects observed by Meli et al., who showed liver damage with inflammatory cell infiltration after 5 weeks, which was intensified after 8 weeks of a HFD [19]. In our previous study on a 5-week animal model, an increase in the total DAG, TAG and FFA concentrations occurred that correlated with the observed changes in the liver histology. These results suggested a relationship between an excessive accumulation in the cytoplasm of hepatocytes, mainly as triacylglycerols, and the development of NAFLD [13]. The imbalance between the amount of supplied fatty acids and the ability of the liver to store them in the form of lipid droplets prompted us to verify changes in the expression of enzymes that catalyze the esterification of diacylglycerol to TAG [20]. Therefore, we focused on changes in these enzymes’ expression accompanying the increased lipid accumulation observed during NAFLD progression [21]. In our study, we observed a significant increase in DGAT1 expression only in the first 3 weeks, and at the next time points (fourth and fifth weeks), these values decreased. Probably, the observed increase in DGAT1 is one of the possible protective mechanisms of the liver against increased fat bioavailability. Thus, we suspect that its elevated expression may intensify the FFAs’ esterification to the TAGs, which may be stored mainly in the liver and adipose tissue, leading to increased total body weight as a symptom accompanying NAFLD. Likewise, research conducted in the same animal model showed a linear increase in the liver’s TAGs [13]. In the last 2 weeks of our study, the DGAT1 expression decreased. It is possible that this may be a response to the intensified hepatic secretion of TAGs into the bloodstream that is intended to prevent the excessive accumulation of lipids in this organ. The confirmation of this hypothesis may be the fact that after HFD administration, in rat and mouse models, an increased expression of proteins involved in the transport of fatty acids out of the cells into the circulation, i.e., microsomal triacylglycerol transfer protein (MTP) and ATP-binding cassette transporter A1 (ABCA1), was observed [13,22]. Moreover, visible changes in the expression of hepatocyte-specific DGAT2, an enzyme that uses *de novo*-synthetized fatty acids to form TAGs, are in line with the results obtained by Liu et al. These findings proved that in the liver, elevated DGAT2 expression occurred after each time point, indicating the hepatic ability to accumulate TAGs [23]. The greatest change in the DGAT2 expression was observed in the first week of our study, which suggested a liver response to a sudden availability of FFAs and subsequent esterification into TAGs.

High-fat feeding, due to the higher energy availability, leads to changes in the level and composition of fatty acids. Research conducted by Liu et al. showed that after 3 weeks of a HFD, in the serum TAG and FFA fractions, there was a shift in the balance between n-6 and n-3 toward n-6 PUFAs. These results implied that after high-fat chow administration, a rise in PUFAs was largely associated with a pronounced elevation of arachidonic acid and also linoleic acid, an AA precursor [24]. Moreover, the changes in hepatic PUFA composition were the result of increased lipid oxidation and also membrane peroxidation of PUFAs [25,26]. These findings are consistent with our observations where we noticed increased AA levels in the examined hepatic lipid fractions. In line with our results, the studies conducted by Ma et al. showed a significant increase in the total AA concentration in the liver at weeks 4 and 8 of the HFD. What is more interesting is that a decrease in AA in the following weeks of the high-fat feeding model in NAFLD (12 and 16 weeks) was observed [27]. It is probable that the reduced arachidonic acid concentration at the end of study reflected the liver’s adaptation to chronic inflammation, and the inflammatory changes were more severe on the 50th day than on the 30th day of the experiment [28]. However, we paid attention to the short-term feeding and focused exclusively on the changes that occurred during every week of 5-week high-fat chow administration. According to the research by Hall et al., a dietary mice model with NAFLD showed an increase in pro-inflammatory eicosanoids such as n-6 PUFA metabolites. Furthermore, they confirmed that the phospholipid membrane, as a source of substrates for free AA, contributes to the development of inflammation and finally cell destruction. A HFD resulted in the enhanced release of AA from membrane phospholipids involved in the generation of eicosanoids and positively correlated with an increase in the activity of inflammatory pathway enzymes [29]. After 1 and 16 weeks of high-fat feeding, mice exhibited an increase in hepatic arachidonic acid levels in all the lipid fractions. Interestingly, the elevation of AA in the PL fraction was already significant by the first week of the HFD and steadily increased in the following weeks of the experiment [30]. These reports are in accordance with the increase in the arachidonic acid concentration in the liver phospholipid fraction observed herein. We suspect that elevated AA levels occurring especially in the PL, FFA and DAG fractions are the first step in the development of inflammation after the first week of our experiment and that further increased AA levels are the most important source of inflammatory precursors. Lipid droplets formed in the hepatocytes are composed of TAGs surrounded by phospholipids that are also a source of enormous levels of AA derivatives. Consistent with this notion, we also observed an increased expression of the enzymes participating in the synthesis of leukotrienes and prostaglandins [2,31]. The higher AA amounts display pro-inflammatory properties, enhancing the inflammation signaling cascade. Lipid pro-inflammatory mediators are generated in the arachidonic acid catabolism pathway catalyzed by cyclooxygenase and lipoxygenase [18,31,32]. Tsujimoto et al. reported that increased COX-1 and COX-2 gene expression in mice fed with a HFD for 12 weeks was associated with hepatic steatosis development. We suspect that the increase in the expression of these enzymes in the liver homogenates observed in our study is similar to the changes in hepatic mRNA expression observed in Tsujimoto et al.’s research [33]. Furthermore, previous research conducted on a rat model during a HFD showed an increase in NF-κβ and a decrease in Nrf-2 hepatic expression [34]. These findings are in line with our observations. The lower expression of the transcription factor Nrf-2 is probably a consequence of the disturbance of lipogenic and cholesterologenic pathways due to increased lipid accumulation and the development of oxidative stress. The altered expression of Nrf-2 resulted in disorder in important antioxidant gene expression regulation, leading to the disruption of antioxidant protective mechanisms. In the case of NF-κβ, our study reported that the consumption of the HFD promoted the activation of this factor, which stimulated the expression of matrix and chemotactic proteins’ genes involved in the production of pro-inflammatory cytokines that may be the main inducer of inflammation development [34,35]. The precise role of IL-6 in NAFLD seems to be controversial. Activated cytokine production participates in a protective role in HFD-induced liver fibrosis. Increased IL-6 expression promotes hepatocyte proliferation and also protects against the redox imbalance. On the other hand, there are some studies showing that IL-6 is a potential inflammatory mediator in NAFLD development and that the hepatic expression of IL-6 positively correlates with the stage of liver inflammation [21,36,37]. In the last weeks of the experiment, we observed a trend toward to an increased expression of NF-κβ and IL-6, which could significantly increase over a prolonged period of fat feeding. Experiments conducted on the following models—rats, for NF-κβ, and mice, for IL-6, with 12 and 7 weeks of a HFD, respectively—showed that at the end of these time points of feeding, the animals showed a statistically significant increase in the expression of hepatic NF-κβ and IL-6 [34,38]. We can hypothesize that a longer period of high-fat feeding could cause visible changes in the examined parameters that may deteriorate steatosis to NASH. To definitively address the question of when NAFLD deterioration to NASH occurred, we investigated oxidative stress pathway parameters. There are studies that showed a high level of oxidative stress characterized by the depletion of antioxidants that resulted from elevated hepatic fat deposition [39]. Lipid accumulation, mainly in the FFA fraction, may contribute to the mitochondrial dysfunction that involves the impairment of β-oxidation, the TCA cycle and the respiratory chain [40]. The result of these impairments is an increased generation of reactive oxygen species (ROS), thus enhancing oxidative stress and NAFLD deterioration. Moreover, intensified lipid peroxidation, especially of membrane PUFAs, can initiate the formation of bioactive metabolites, such as MDA, which, through sustained lipotoxicity and the altered lipid signaling of membrane phospholipids, may cause NAFLD progression to NASH [4]. Our observations included elevated concentrations of lipid peroxidation by-products, such as MDA. Moreover, we observed a trend toward to an increase in the concentrations of lipid breakdown products, such as 4-HNE, and suppose that these phenomena can induce significant changes over a longer period of high-fat feeding. On the other hand, in our experiment, the rats fed the HFD showed a reduced content of antioxidant enzymes. The SOD2 level had already decreased in the first week of the experiment, which may be the liver’s response against the sudden increased fat administration. Additionally, a decrease in the CAT level was most noticeable in the fifth week of our study. In the last three weeks of our experiment, we observed a slight increase in the total antioxidant capacity. Supporting these data are studies conducted by Tao et al., where a slight rise in the TAC after 7 weeks on the HFD in a mouse model was observed. It also may be implied that such changes express the liver’s defense mechanism against oxidative imbalance by increasing the antioxidant capacity [38]. Importantly, the concentration of AGEs had already increased rapidly in the first week of the HFD, which is a response to the increased availability of lipids and is reflected by the emerging lipid droplet deposition in the liver at the same time. More surprisingly, AGEs significantly decreased in the last week of the study. We suspect that the decline may be associated with the liver microvasculature dysfunction provoked by the impaired metabolism of AGEs. Hepatic impairment may cause an increased efflux of these non-enzymatic reaction products into the bloodstream, a situation that is observed in the sera of patients with NAFLD [41].

## 5. Conclusions

**Scheme 1:**
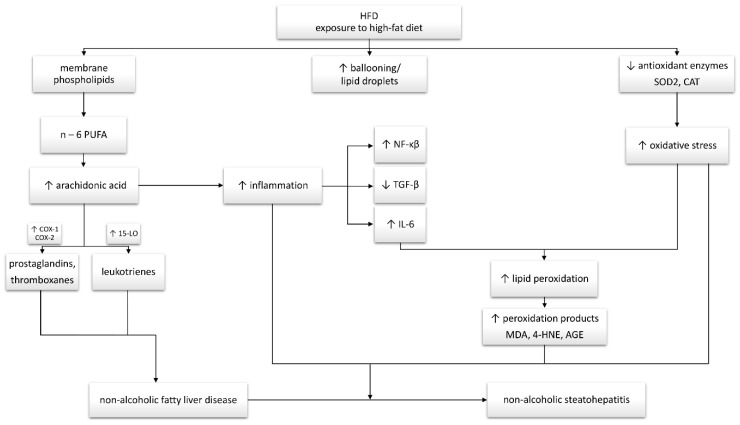
Interplay between the hepatic arachidonic acid level, inflammation and oxidative stress in non-alcoholic fatty liver disease (NAFLD) development and progression to non-alcoholic steatohepatitis (NASH). Changes in the examined parameters are indicated on the diagram.

In summary, the study clearly suggests that high-fat feeding promotes the excessive accumulation of arachidonic acid as a pro-inflammatory precursor. Of particular importance is the linear increase in the concentration of AA, mainly in the PL fraction, excessively accumulated in the liver. The observed AA changes and increased expression of enzymes involved in eicosanoid and prostanoid production are consistent with the development and severity of inflammation during NAFLD development. Moreover, our results indicated a rapid development of inflammatory precursors as early as the first week of high-fat feeding. We demonstrated a significant HFD impact on the development of oxidative stress with a simultaneous impairment of the antioxidant system. This has been confirmed by the observed increase in lipid peroxidation products with a simultaneous change in the content of oxidative stress pathway enzymes and TAC. Based on the observed changes in the arachidonic acid concentration, increase in COX-1 and 15-LO expression and oxidative modifications, we may suspect that HFD-induced pro-inflammatory disorders in the liver start as early as in the first week of HFD challenge (Scheme 1). Moreover, these parameters can be potential prognostic markers of NAFLD development in the early stages of this disease.
